# Cognition‐Enhanced Machine Learning for Better Predictions with Limited Data

**DOI:** 10.1111/tops.12574

**Published:** 2021-09-16

**Authors:** Florian Sense, Ryan Wood, Michael G. Collins, Joshua Fiechter, Aihua Wood, Michael Krusmark, Tiffany Jastrzembski, Christopher W. Myers

**Affiliations:** ^1^ InfiniteTactics LLC; ^2^ Department of Experimental Psychology University of Groningen; ^3^ Behavioral and Cognitive Neuroscience University of Groningen; ^4^ Department of Statistics University of Oxford; ^5^ Air Force Research Laboratory Oak Ridge Institute for Science and Education; ^6^ Department of Psychology Wright State University; ^7^ Ball Aerospace & Technologies Air Force Research Laboratory; ^8^ Department of Mathematics and Statistics Air Force Institute of Technology; ^9^ L3Harris Technologies at Air Force Research Laboratory; ^10^ Air Force Research Laboratory

**Keywords:** Cognitive model, Machine learning, Prediction, Memory, Learning, Gradient boosting

## Abstract

The fields of machine learning (ML) and cognitive science have developed complementary approaches to computationally modeling human behavior. ML's primary concern is maximizing prediction accuracy; cognitive science's primary concern is explaining the underlying mechanisms. Cross‐talk between these disciplines is limited, likely because the tasks and goals usually differ. The domain of e‐learning and knowledge acquisition constitutes a fruitful intersection for the two fields’ methodologies to be integrated because accurately tracking learning and forgetting over time and predicting future performance based on learning histories are central to developing effective, personalized learning tools. Here, we show how a state‐of‐the‐art ML model can be enhanced by incorporating insights from a cognitive model of human memory. This was done by exploiting the predictive performance equation's (PPE) narrow but highly specialized domain knowledge with regard to the temporal dynamics of learning and forgetting. Specifically, the PPE was used to engineer timing‐related input features for a gradient‐boosted decision trees (GBDT) model. The resulting PPE‐enhanced GBDT outperformed the default GBDT, especially under conditions in which limited data were available for training. Results suggest that integrating cognitive and ML models could be particularly productive if the available data are too high‐dimensional to be explained by a cognitive model but not sufficiently large to effectively train a modern ML algorithm. Here, the cognitive model's insights pertaining to only one aspect of the data were enough to jump‐start the ML model's ability to make predictions—a finding that holds promise for future explorations.

## Abstract 2

Cognitive models and machine learning techniques were combined to improve predictions in naturalistic vocabulary learning data. Key predictive features for the ML model were distilled from the cognitive model to better capture the underlying temporal dynamics.

## 1 Introduction

With limited multidisciplinary cross‐talk between the machine learning (ML) and cognitive science communities, predictive analytics research is overly stovepiped. Both fields of research have adopted standard methodological paradigms to suit their needs, each possessing their own strengths and weaknesses. However, by integrating ML and cognitive science methodologies, there is an opportunity for each respective discipline's strengths to be exploited and weaknesses to be remedied (Griffiths, 2015; Mozer & Lindsey, 2016; Sense, Jastrzembski, Mozer, Krusmark, & van Rijn, 2019). Successful integration of these approaches could result in enhanced predictive power, minimized data requirements, and deeper theoretical understanding across a wide range of domains. One highly relevant domain and the focus of this paper is human learning.

The success of statistical ML models in diverse applied settings stems from their ability to identify relationships across multiple noisy inputs. However, such models typically require access to large, curated/annotated datasets to make high‐quality predictions (Hastie, Tibshirani, & Friedman, 2009), and often constitute “black boxes” with (sometimes) millions of uninterpretable parameters. Consequently, it becomes near impossible to determine why models make the decisions they do (Gunning, 2017).

Cognitive scientists, on the other hand, develop and implement theory‐driven models capable of explaining and interpreting human empirical data (McClelland, 2009). Such models usually have a fixed mathematical structure representing specific theoretical assumptions and ideally capture input data variations through a limited number of free parameters that map onto psychological measurements and cognitive processes. An advantage of this approach is that less data is required to fit models. Conversely, these models are often rigid and usually unable to incorporate additional (meta‐)data not anticipated by the theoretical model (e.g., future performance *only* depends on past performance and its exact timing) and they generalize poorly to noisy or naturalistic domains because they are rarely evaluated on their ability to make quality out‐of‐sample predictions (Yarkoni & Westfall, 2017).

Recent work by Riesterer, Brand, and Ragni (2020) nicely illustrated the different approaches. They compared various cognitive models’ ability to fit human data from a syllogistic reasoning task. The authors fit three neural network models to obtain an “upper limit” of statistical regularity in the data. Showing that the neural networks outperform any cognitive model they tested, the authors conclude that there was room for improvement. Notably, the ML techniques were used to separate the signal from the noise in the data—they were not assumed to inform our understanding of how humans solve syllogistic reasoning problems. The current work follows recent efforts to bridge this gap by building cognition‐inspired ML models (e.g., Mozer & Lindsey, 2016; Settles & Meeder, 2016; Trafton, Hiatt, Brumback, & McCurry, 2020). Recent advances in leveraging cognitive insights in large‐scale ML in the domain of human decision making (Bourgin, Peterson, Reichman, Russell, & Griffiths, 2019; Peterson, Bourgin, Agrawal, Reichman, & Griffiths, 2021) illustrate the utility and promise of this approach particularly well.

### 1.1 The current study

We are interested in potential ways to combine the strengths and mitigate the weaknesses of ML and cognitive modeling. The current work is a first exploration of this endeavor. Our central research question was whether insights from a cognitive model could be leveraged to enhance the predictive accuracy of an ML model. To start, we identified a promising large‐scale, naturalistic dataset to assess our integrated approach against. Second, we developed specific implementations of an ML model and a cognitive model of learning and retention for integration.

The dataset we selected was from a naturalistic task in the domain of language learning. This dataset was from the 2018 Second Language Acquisition Modeling (SLAM) challenge organized by Duolingo (Settles, Brust, Gustafson, Hagiwara, & Madnani, 2018). Duolingo published learning data spanning the first month of new users on their platform (see the *Dataset* section below for details) and challenged the international research community to submit models that could predict user accuracy on withheld data.

For the ML model, we chose a gradient‐boosted decision tree (GBDT; Friedman, 2001). GBDTs are available in multiple out‐of‐the‐box implementations that are readily deployed and perform well on a wide range of prediction tasks (Bentéjac, Csörgő, & Martínez‐Muñoz, 2020). They are generally used to model tabular data and several top teams in the 2018 SLAM challenge also utilized decision tree ensembles (see table 2 in Settles et al., 2018).

For the cognitive model, we chose the predictive performance equation (PPE). The PPE was developed as a knowledge tracing model that could account for the nonlinear, multiplicative effects of learning and forgetting over time, with a particular focus on predicting future performance (Jastrzembski, Gluck, & Gunzelmann, 2006). We argue that the PPE should be directly relevant to the Duolingo data because spacing effects assume a central role as users on the platform learn (and forget) new materials over time (Settles & Meeder, 2016). A detailed description of the PPE, its theoretical foundation, comparison to other alternative cognitive models, and applied potential are documented elsewhere (Walsh et al., 2018, Walsh, Gluck, Gunzelmann, Jastrzembski, & Krusmark, 2018). In short, the PPE is a set of nested equations that estimate the activation *M* of an item in human memory:

(1) $$M=N^{c}*T^{-d}.$$

Activation is the product of a learning term, *N^c^*, and a forgetting term, *T^–d^*. Within the forgetting term, the temporal dynamics of forgetting are captured by model time, *T*, and the decay rate, *d*. Model time is the weighted sum of time elapsed (*t*) for each training repetition (*i*):

(2) $$T=\sum_{i=1}^{n}w_{i}*t_{i}.$$

The weights *w_i_* assigned to each repetition decrease with elapsed time *t*:

(3) $$w_{i}=\frac{t_{i}^{-x}}{\sum_{j=1}^{n}t_{i}^{-x}}.$$

The parameter *x* is set to a default value of 0.6. The model time term isolates the information in the learning history which describes the age of items in memory. Since learning happens over many instances, all of them are considered relevant to the learner's future performance. However, the importance of these instances should be skewed toward the most recent ones (Walsh et al., 2018).

The decay rate captures the phenomenon that spaced practice produces more stable learning than massed practice (Dempster, 1988):

(4) $$d_{n}=b+m\cdot \left(\frac{1}{n+1}\cdot \sum_{j=1}^{n-1}\frac{1}{\ln(lag_{j}+e)}\right).$$

Intervals between training repetitions are scaled such that long lags produce a smaller decay rate, while shorter intervals produce a larger decay rate. Combined with an additive and multiplicative constant, optimized over training data, the decay rate extracts the essence of the “spacing effect” from the full history of lags (Pavlik & Anderson, 2008; Walsh et al., 2018). The component of Eq. 4 in parentheses is called the *stability* term and does not depend on estimated parameters; it is a direct transformation of raw lag time.

Both choices of models—the GBDT and the PPE—represent state‐of‐the‐art modeling approaches in their respective domains and were selected because they were expected to be particularly suitable to modeling performance in the chosen task domain. The goal of the current study was to assess whether providing the cognitive model's summary of a learning history to a state‐of‐the‐art ML model would improve predictive accuracy. Specifically, the information contained in the training history that is most relevant to predicting retention—model time and stability—before the ML model is trained. This restricts the feature space of the dataset, which might make it easier for the ML model to search for the best prediction function. This “smoothing” should be beneficial if the cognitive model's mechanism captures relevant statistical regularities in memory over time. We expect that both of these potential benefits would emerge depending on how much data is available to train the model.

With sufficient data, the GBDT will learn the optimal mapping with or without the cognitive insights from PPE. With limited amounts of training data, however, transforming the raw timing data using PPE's assumptions about underlying human memory processes is expected to yield benefits to the GBDT. Consequently, we present explorations of various restricted data scenarios and demonstrate that, indeed, a state‐of‐the‐art ML model's predictive validity is enhanced when it leverages the insights of a cognitive model.

In the following sections, we will detail the methods of our approach, focusing on the dataset used and the specific implementations of our modeling approach. We will then present the results of our explorations, highlighting the conditions under which the PPE‐enhanced model might be most advantageous. And finally, we will conclude by discussing our findings and presenting implications for future work.

## 2 Methods

In this section, we describe the dataset used for our explorations in more detail, and provide details on the models and model fitting procedures. For additional details, see the descriptives in the online Supplement at https://osf.io/54ry7/.

### 2.1 Dataset

For the current explorations, we used the dataset Duolingo released for the 2018 SLAM challenge (Settles et al., 2018). These data contain learning histories from three languages over a 30‐day period.^1^ Specifically, they “sampled from Duolingo users who registered for a course and reached at least the tenth row of skill icons within the month of November 2015. By limiting the data to new users who reach this level of the course, we hope to better capture beginners’ broader language‐learning process […]” (Settles et al., 2018, p. 56). The data from each of the three language tracks are entirely separate. We focused on the English track for our current analyses, as this track represented the largest subset of data. The user's task was to accurately respond to a range of language learning exercises that included various forms of translation and listening (see fig. 1 in Settles et al., 2018).

**Fig 1:**
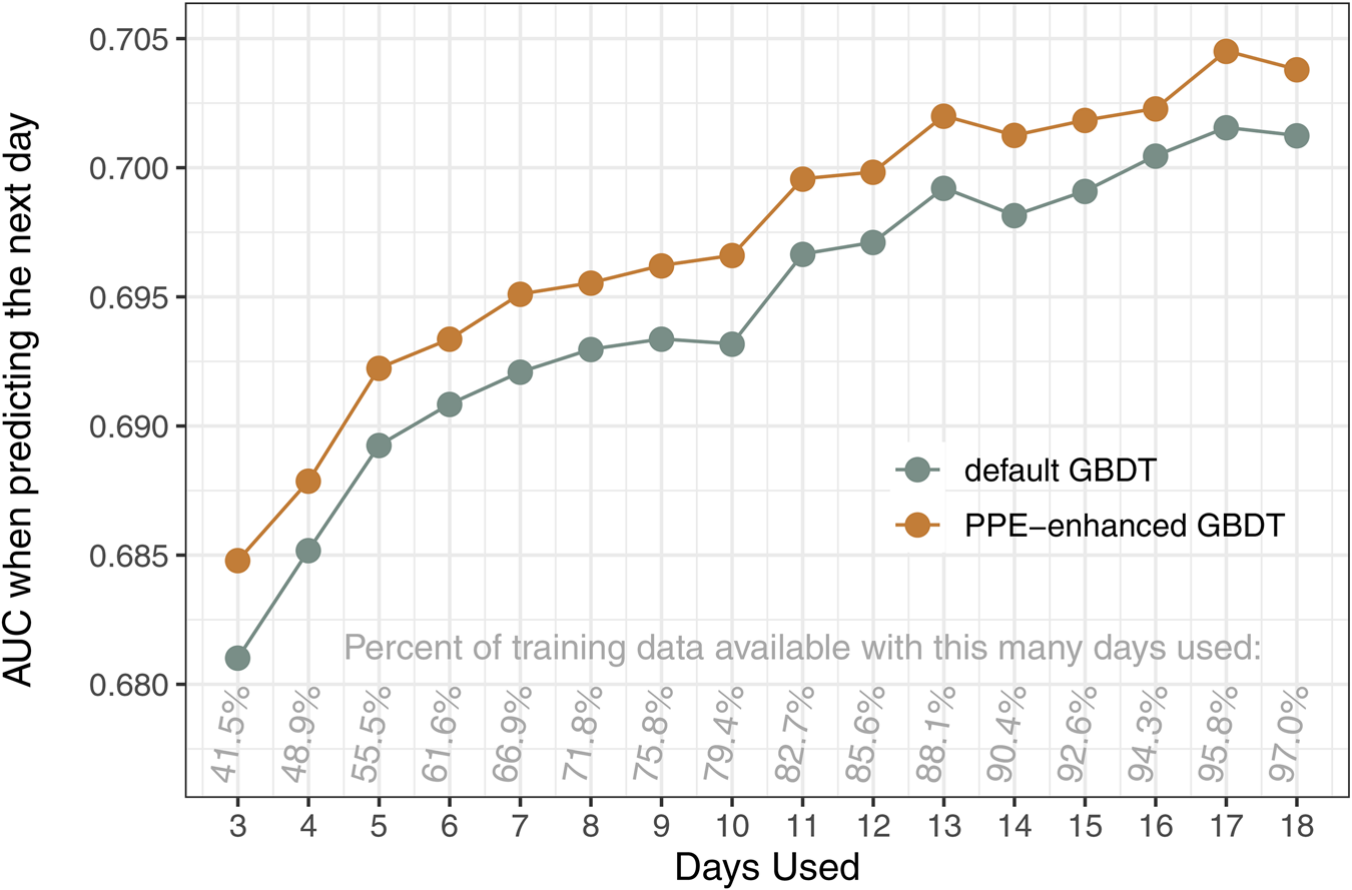
AUCs of the two models in the restricted learning history scenario. The days used on the *x*‐axis indicate how much of the training data was used to train the models; the AUCs are based on predicting accuracy on the next (*x*+1) day.

We fit the two models outlined in the *Models* section below to (1) the full dataset published by Duolingo, and (2) specific slices of the dataset. We will describe both in turn.

#### 2.1.1 The full dataset

For the challenge, Duolingo released the data in three phases.^2^ The first phase of the challenge afforded training on the first 80% of the data (TRAIN set). The second phase came with a release of the next 10% of the data (DEV set), for which participants could evaluate and fine‐tune their models. Finally, the third phase allowed for predictions on the remaining 10% of the data to be submitted (TEST set). The task, as set out by Duolingo, was to predict which tokens, defined as distinct user‐item pairs, users answered incorrectly in the TEST set.

In the published challenge, the user's true performance was omitted from the TEST set and was not available on the website. As a result, we were not able to evaluate our modeling efforts on the same TEST set used in the SLAM challenge. To remedy this without taking an unfair advantage over participants in the original competition, we combined the TRAIN and DEV sets and assigned the first 90% of instances for each learner to the *train* set, while the last 10% were assigned to the *test* set. This way, we emulated the SLAM challenge as closely as possible.

The *full dataset* used here is thus based on 90% of the complete SLAM dataset published by Duolingo and follows a 90/10 train/test split. The training set contains 2,347,874 observations from 2593 users studying 2164 tokens (745,459 distinct user‐token pairs). Accuracy in the training set is high (87.6% overall), increasing as a user repeats a token (from 83.5% on the first repetition and plateauing around 91% after the eighth repetition). However, repetitions are rare: 49% of user‐token pairs are only repeated once (9% were repeated more than five times) and 71.8% only on a single day. The test set contains 275,083 observations from the same 2593 users responding to 1925 tokens (153,008 distinct user‐token pairs). Accuracy in the test set was similarly high at 85.8% and two‐thirds of the user‐token pairs were only repeated once (3.9% were repeated more than five times).

#### 2.1.2 Slices of the dataset

To investigate the potential benefits of enhancing an ML model with insights from a cognitive model, we explored two different ways of limiting the amount of information available to train the models. Thus, the approaches differ in what slice of the dataset was used, both with regard to the training and test sets. Furthermore, we decided to use only a limited set of input features that would be available for any comparable dataset (user, token, and timing information). This will impair the model overall but isolates the effects that are most generalizable and important (Table 1).

**Table 1:** Normalized feature importance of both models’ top 10 features

Default GBDT		PPE‐enhanced GBDT	
Input feature	Feature importance	Input feature	Feature importance
User	21.3%	Token	23.8% (+5.0%)
Token	18.8%	Format	13.3% (+2.5%)
Format	10.8%	Seconds	8.8% (+1.1%)
Seconds	7.7%	User	6.2% (–15.1%)
Prompt	6.9%	Prompt	6.0% (–0.9)
Root dependency	5.8%	Root dependency	5.1% (–0.7%)
Lag 1	4.6%	Days	4.0% (+2.0%)
Next token	3.6%	Exercise total tokens	2.9% (—)
Previous token	2.6%	Next token	2.8% (–0.8%)
Days	2.0%	Token length	2.2% (—)

Note: Numbers in parentheses are the change in normalized feature importance going from the default to the PPE‐enhanced GBDT.

The training set was sliced to limit the available data in two ways: First, by gradually exposing the models to more data day‐by‐day. In this *restricted learning history* approach, we made iterative predictions by fitting both models to the data up to day *x* and then making and comparing predictions made for day *x* + 1. The second approach sought to limit the amount of information available to train the models by *restricting the number of users and tokens* included in the training set. This was achieved by randomly sampling *n* ∈ {5, 10, 20, 50, 100, 250, 500} users/tokens from the training set and fitting both models to that slice of data. One hundred iterations were run for each of the 49 combinations.^3^

The test set was always subset to only include the first repetition of tokens that appeared in the training set the to‐be‐evaluated model was trained on. This decision was based on two considerations: First, in most applied settings, predictive models used for adaptive learning software would only predict performance on the next exposure and then take the actual response into account to calibrate the prediction for the subsequent prediction (Lindsey, Shroyer, Pashler, & Mozer, 2014; Pavlik & Anderson, 2008; Sense, Behrens, Meijer, & van Rijn, 2016). And second, making a prediction for a novel token is not a task a cognitive model would typically be leveraged for (collaborative filtering/recommender systems are better suited for this task, Su & Khoshgoftaar, 2009).

For all approaches based on slices of the dataset, we simplified the model by using only a small subset of input features, namely user and token identifiers, and timing information (expressed in days). These turned out to be the most important features (Table 1). Regardless of their importance, we believe that these are features that would be available in virtually all comparable prediction tasks in the context of language learning. Hence, restricting our explorations to a small number of near‐universal features hopefully makes the results more generalizable.

### 2.2 Models

For all approaches and subsets of the dataset outlined above, we always fit two models. Their predictive accuracies were evaluated using the area under the receiver operating characteristics curve (AUC; Fawcett, 2006). The two models evaluated in this paper were GBDT and differed only with regard to how the timing related information was made available as input features. Both models had access to the “raw” timing information. The *default GBDT* had 10 additional input features that explicitly coded the lag (elapsed time) since the last, second‐to‐last, and so on repetition for each user‐token pair. The *PPE‐enhanced GBDT* only had two additional input features: The model time *T* and the stability component of the decay rate equation (see Eqs. 2 and 4, respectively). The PPE transformations imposed theoretical assumptions about how memory traces accumulate and decay over time. Thus, the reduction in input feature dimensionality was paired with a biased preprocessing of the timing information. Importantly, however, both models have access to the same raw timing information; no new information is added. Hence, the only difference between the models is the inductive bias provided by PPE. Our core research question, as outlined above, is whether and/or under which circumstances such bias might be beneficial to a state‐of‐the‐art ML model.

#### 2.2.1 Model fitting

Each *instance* is defined by the SLAM competition as one word within a translation problem. Each timestamped instance is coded as a 0 if the word was translated correctly and a 1 if the word was translated incorrectly by the Duolingo learner. Due to its efficiency, accuracy, and ability to use high‐cardinality categorical features, the LightGBM^4^ implementation of gradient boosting (Ke et al., 2017) was chosen as the model for our binary classification problem. LightGBM's ability to leverage high‐cardinality categorical features is particularly useful because a number of the (important) predictors are categorical variables with many levels (user, token, and prompt; see Table 1 and online Appendix A at https://osf.io/fj8mr/). The parameter values were optimized on a validation set derived from the train set. Cross validation was not used because of the temporal nature of the data. We used the same optimized parameters for all model fits reported here.^5^

## 3 Results

To evaluate the default GBDT and the PPE‐enhanced GBDT, we will report the area under the ROC curve (AUC; Fawcett, 2006) to quantify a model's predictive accuracy. AUC values range from 0.5 (chance performance) to 1.0 (perfect predictions) and were chosen because they are widely used as a measure of predictive accuracy in binary classification tasks and, specifically, they were the primary outcome measure in the SLAM challenge. The raw data and analysis scripts are available in an online Supplement at https://osf.io/vk8jr/, which contains the code to reproduce all numbers and figures reported here along with additional descriptives and visualizations. The rest of this section will follow the distinction made in the previous section and present the outcomes of our explorations, first on the full dataset and then on slices of the dataset.

### 3.1 Full dataset

The two GBDTs were fit to the complete training set using both the provided and additionally engineered features (see online Appendix A at https://osf.io/fj8mr/). For the default GBDT, the AUC for the predictions was 0.8530, which compares favorably to the published models reported in Settles and colleagues (2018, table 2)—slightly lower than the top three AUCs, which were 0.859 and 0.861 (tied), and comfortably outperforming the baseline (0.774). The PPE‐enhanced GBDT resulted in a slightly higher AUC of 0.8538.

The correlation between the two models' predictions is extremely high (*r* = 0.985) and a Bayesian paired *t*‐test suggests the data are 17.6 times more likely to occur under the null model (Morey & Rouder, 2018). However, DeLong's test reveals a statistically significant difference between the AUC values (*z* = –3.42, *p* < .001). Thus, the two models’ predictions are very similar but produce slightly different AUC values. Therefore, the GBDT is not impaired when PPE's terms are used as input features. In fact, predictions are slightly better; but the difference, albeit statistically significant, is probably too small to be practically relevant.

Table 1 lists the feature importances normalized as percentages across all features for each model. A feature importance of X percent implies that X percent of the reduction in impurity is attributable to decision tree splits on that feature. Impurity here generally refers to any measure of how many incorrectly classified observations exist in a set of training data. Popular examples of impurity measures include misclassification rate, entropy, and the Gini index (Hastie et al., 2009, pp. 309–10). In this case, we used the Gini index for the measure of impurity (Breiman, Friedman, Stone, & Olshen, 1984).^6^ The ranking of the features in Table 1 suggest that in order to make accurate predictions in this task, the exact timing of practice is not nearly as important as who is expected to give a response to which token in which context (i.e., prompt, previous, and next token).

Taken together, the analyses on the full dataset suggest that the PPE‐enhanced model performs at least as well or marginally better even though the dimensionality of the input is reduced relative to the default GBDT. Hence, providing the GBDT with PPE's preprocessed timing information did not impair the model. It appears that both GBDTs made very similar but not identical predictions that slightly favored the PPE‐enhanced model. Surprisingly, timing‐related information did not appear to be as important as we anticipated (Table 1). Next, we explore conditions under which the PPE‐enhanced model could have a more noticeable advantage.

### 3.2 Slices of the dataset

In the following, a number of scenarios are explored in which the data used to train the two models were restricted. These slices of the dataset were designed specifically to yield conditions that might be favorable to a cognitive model (see *Methods* above).

#### 3.2.1 Restricted learning history

To isolate the effects of having a more extensive learning history available when making predictions, we utilized a step‐wise prediction approach. The AUCs for the day‐by‐day predictions are shown in Fig. 1, which also indicates the percentage of the training data that were used on each day. Past day 18, less than one percent of training data were added, which leaves very few observations to predict with this iterative approach, resulting in unreliable and highly variable AUCs. Hence, we omitted the last week of data from the figure (see the online Supplement for additional visualizations of the distributions of observations/user/tokens over time).

Fig. 1 shows that the PPE‐enhanced GBDT has a small but consistent advantage over the default GBDT. Predictions generally become better with more training data but since the increase of data over days is not linear (see the percentages noted in the graph), the steepest improvement in AUCs is achieved early on. The absolute difference in AUCs is fairly stable but since the AUCs increase overall, the relative advantage is slightly larger with a more restricted learning history.

#### 3.2.2 Restricting number of users and tokens

**Fig 2:**
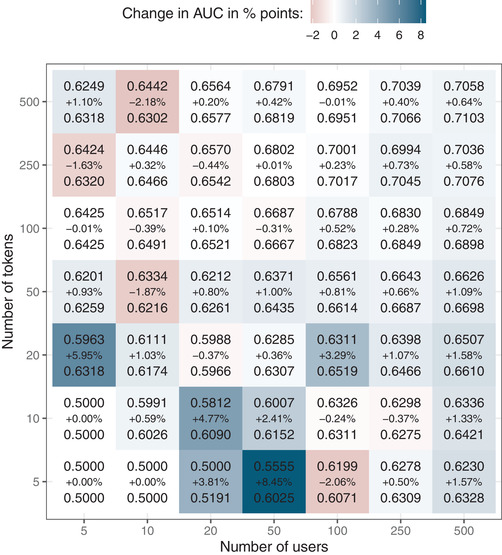
AUC differences when the number of users and tokens are restricted. The data are subset by randomly sampling *x* users and *y* tokens. Each cell summarizes the averaged results by listing the median AUC of the default GBDT (top), the AUC of the PPE‐enhanced GBDT (bottom), and the change from the former to the latter in percentage points (middle). The color coding is based on the change in AUC to highlight under which conditions the PPE‐enhanced model performs best.

Besides restricting the learning history along the temporal dimension, we also explored limiting the amount of data available to train the models. Both models were trained on 100 samples for each combination of user/token pairings we explored (see *Methods* for details). This yielded a set of predictions on the test set for each model, from which AUC scores were calculated. The results are summarized in Fig. 2, which shows in each cell the median AUCs for the default and PPE‐enhanced GBDT (top and bottom values, respectively) as well as the percentage change.

We see an overall advantage of the PPE‐enhanced model with more data. In the lower left quadrant of Fig. 2, the advantage of the PPE‐enhanced GBDT is markedly larger relative to the default GBDT, with various user‐token combinations resulting in advantages of 3–8% points. When both the number of users and the number of tokens are larger, advantages rarely exceed 2% points. With 100+ users and tokens, the PPE‐enhanced model's small advantage is consistent. Across the board, the average AUC of the default GBDT is 0.6397 and that of the PPE‐enhanced GBDT is 0.6432, a difference 81.5 times more likely under a model that assumes unequal means, according to a Bayesian paired *t*‐test (Morey & Rouder, 2018). Fig. 2 suggests that this overall difference is primarily driven by the PPE‐enhanced model's superior performance when more users are added to the training set; simply having more tokens but very few users generally favors the default GBDT. The online Supplement includes additional figures that show the distribution of AUC values in each cell of Fig. 2.

**Fig 3:**
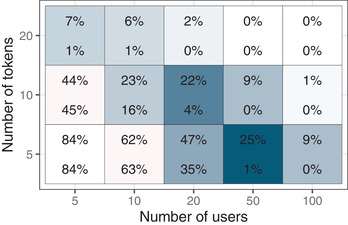
Percent of samples with predictions at chance level. Each cell shows the percentage of samples that yielded AUC values of 0.5 for the default GBDT (top) and PPE‐enhanced GBDT (bottom). The color gradient indicates the magnitude of the difference within a cell.

As expected, there is also a general increase in AUCs as the number of both users and tokens increases. Conversely, we see poor predictive accuracy with very limited data. Since the cells in Fig. 2 show the median AUCs, we can conclude that at least half the models with the least amount of training data perform at chance‐level (i.e., AUC = 0.5). Fig. 3 zooms in on the lower left corner of Fig. 2 and depicts, for each cell, the percent of samples for which the default (top number) and PPE‐enhanced (bottom number) GBDT produce at‐chance predictions. In cells not shown in this figure, both models always produce above chance predictions.

Taken together, we can conclude that there is a consistent advantage of the PPE‐enhanced model and that this advantage was especially pronounced when the amount of training data was limited. Using the PPE transformations of the timing information allowed the GBDT to more quickly overcome chance performance under conditions with extremely limited amounts of data.

## 4 Discussion

Here, we attempted to enhance the performance of an ML model by incorporating insights from a cognitive model. We used second language acquisition data published by Duolingo to compare two GBDTs (Friedman, 2001; Ke et al., 2017)—one using raw timing information and one using cognition‐inspired transformations of said information as input features. These transformations were based on the PPE (Walsh et al., 2018), a cognitive model developed to capture theoretically grounded measures of human learning. When evaluated on the full dataset, the PPE‐enhanced GBDT performed marginally better than the default model but produced largely comparable performance predictions. This suggests that with a sufficiently large dataset, transforming the timing information does not impair the GBDT and may even provide a small benefit.

### 4.1 Cognition‐enhanced ML

In many applied learning domains, however, datasets are not sufficiently large to effectively train state‐of‐the‐art ML models. Since cognitive models are readily fit on small datasets, we expected the PPE‐enhanced GBDT to prove particularly beneficial with limited data. To this end, we pitched the two models against each other in several smaller slices of the full dataset and found that, indeed, the PPE‐based transformation of the timing‐related input features can improve the GBDT's predictions. Specifically, training the models on restricted learning histories impairs predictive accuracy overall, but this impairment is consistently lower for the PPE‐enhanced variant (Fig. 1). Furthermore, restricting the number of users and tokens available in the training set is less debilitating for the PPE‐enhanced model (Fig. 2), primarily because a larger proportion of very poor extrapolations from small training sets are avoided (Fig. 3).

To our surprise, the timing information did not prove to be as important as anticipated (e.g., Ridgeway, Mozer, & Bowles, 2017; Steyvers & Benjamin, 2019). Timing‐related features are near the bottom of Table 1, for example, which suggests that the temporal dynamics only had limited impact on predictions made by our models. This is surprising since earlier work of researchers at Duolingo identified decay over time as an important feature (Settles & Meeder, 2016) and several teams in the SLAM competition explicitly engineered temporal features (e.g., Chen, Hauff, & Houben, 2018; Rich, Osborn Popp, Halpern, Rothe, & Gureckis, 2018).

One potential reason is that many of the tokens were only seen once. Thus, these data points do not constitute a learning history. While this issue is present in this particular dataset due to the instructional learning design of Duolingo itself, it may be nonexistent in many real‐world datasets. We anticipate that the modest improvement in accuracy of the PPE‐enhanced model may actually underrepresent the potential for this technique to improve accuracy on human learning datasets in general, where study repetitions would normally be plentiful. Future extensions of this work should, therefore, explore the approach presented here in other datasets—from both naturalistic and experimentally controlled settings—where repetitions of individual study items are more frequent.

### 4.2 Toward ML‐enhanced cognitive science

The above discussion has focused on the potential benefits of enhancing ML techniques with theoretically grounded insights from cognitive models. We believe that benefits can also be bestowed in the reverse direction: methodologies developed in the statistical learning literature could further extend the reach of theory‐based cognitive models. Such models’ reach is largely determined by their (often implicit) very restricting (and often implicit) assumptions. PPE, for example, gives a theoretically grounded account of performance fluctuations over time. In practice, this means that all changes in performance are a function of time. This deliberate simplification might be sensible in constrained lab settings where the experimenter subsequently manipulates the one dimension of interest (e.g., by imposing predefined study schedules) to learn more about that dimension's influence on performance. In practice, however, there are clearly a number of nontemporal features that influence performance trajectories. ML models provide an excellent means to quantify feature importances explicitly and are not limited to features anticipated by any given theory.

In the current work, we see that the top six features of both models (Table 1) are not related to timing information at all. This information can be used in two ways that can advance our understanding of performance in this domain. First, we can use a cognitive model (assuming proper psychometric properties; Collins, Sense, Krusmark, Fletcher, & Jastrzembski, 2021) and focus on important features to mine the data in theory‐informing ways (Goldstone & Lupyan, 2016). For example, are decay rates estimated for each user‐token combination stable within a *User* or a *Token*? That is, is the difficulty of a given token a function of the token itself or the user's ability and how should that inform our theoretical assumptions? Likewise, one can investigate learning rates as a function of *Prompt* and/or *Format* to inform work on knowledge acquisition and scaffolding (e.g., Kayi‐Aydar, 2013). Granted, these analyses could be conducted without the insights gleaned from Table 1 but we believe that these theory‐agnostic ML methods provide a valuable filter mechanism that highlights to researchers the specific dimensions of a given task or domain that are most in need of an explanation (Goldstone & Lupyan, 2016; Griffiths, 2015; Paxton & Griffiths, 2017).

Second, insights from ML models could be used to go a step further and suggest changes to the structure of cognitive models themselves. For example, in the current work, the response time (seconds) was identified as an important predictor in both models (Table 1; note that the same holds in the analysis of the SLAM challenge results, see Settles et al., 2018). This lends credence to theoretical accounts that link response time to the latent construct of memory activation (e.g., Mettler & Kellman, 2014; Van Rijn, van Maanen, & van Woudenberg, 2009). At its core, PPE aims to trace this latent activation over time (see Eq. 1) and prior work has largely focused on the temporal dynamics. The ML modeling results suggest that it would be valuable to further develop the theory underpinning PPE to explain how aspects such as accuracy and response time (and potentially others) combine into a performance metric that best relates to memory activation.

### 4.3 Conclusion

It is uncommon to have a human learning dataset as vast and as varied as the ones published by Duolingo.^7^ In many applications, the number of users is much smaller and the material set smaller (e.g., an undergraduate class). Such circumstances make it difficult to train powerful ML models but a hybrid, cognition‐enhanced model might be feasible. The work presented here should be understood as but one instantiation of a promising, more general approach: the appropriate cognitive model does not have to be the PPE; the chosen ML method does not have to involve gradient boosting. Using cognitive models to produce features to help train ML models could be applied in other domains.

### Open Research Badges

This article has earned Open Data and Open Materials badges. Data are available at https://dataverse.harvard.edu/dataset.xhtml?persistentId=doi:10.7910/DVN/8SWHNO and materials are available at https://osf.io/vk8jr/.

## Supporting information

Appendix

## Notes

1 For a list of the features available in the dataset (as well as those engineered by us), see online Appendix A at https://osf.io/fj8mr/

2
https://dataverse.harvard.edu/dataset.xhtml?persistentId=doi:10.7910/DVN/8SWHNO

3 Two additional rules were observed to ensure usable slices of the data: (1) The users and tokens were uniformly sampled (without replacement) from the 1000 users and tokens with the most observations. This avoided including users or tokens with very few observations but does bias the analysis slightly toward users that have used Duolingo more and tokens that appear more frequently. Please see the online Supplement for additional descriptive statistics pertaining to this split: https://osf.io/54ry7/ (2) Samples in which the test set did not include at least one correct and one incorrect response were discarded to ensure the AUC can be computed. The online Supplement contains a figure that shows the distribution of sample sizes in the training and test sets, see https://osf.io/yg9ps/

4
https://github.com/microsoft/LightGBM

5 These were: max number of leaves per tree: 50; learning rate: 0.05; minimum number of observations per leaf: 10; and categorical smoothing: 50.

6 Online Appendix B (https://osf.io/fj8mr/) showcases an attempt to visualize the differences between the models by approximating the models with decision trees. This could be another way to illustrate feature importance but we believe it is less informative in the current context.

7 See the section *Data & Tools* at https://research.duolingo.com/
