# Supplementary material for: Cognition‐Enhanced Machine Learning for Better Predictions with Limited Data
Source: Top Cogn Sci. 2021 Sep 16;14(4):739–55. doi: 10.1111/tops.12574 (PMC9786646; doi:10.1111/tops.12574)
Supplement: Supplementary file 1 — Appendix [file TOPS-14-739-s001.pdf]

APPENDIX to
"Cognition-Inspired Machine Learning for Better Predictions with Limited Data"

Appendix A: Input features and feature engineering

We kept many features as they were given in the Duolingo dataset, but derived several more columns based on the given features. Our approach to feature engineering was to add features that provide information relevant to the task of the learner. Among the engineered features was the total number of words in an exercise, the next and previous words of an instance, the number of days since the last times a user encountered the word, the average difficulty of the surrounding words, word length, and the accuracies achieved by the same user on the same word in previous instances.

We chose to keep all other provided features (see http://sharedtask.duolingo.com/2018.html for additional details), which were:

1. User identifier
2. Token identifier
3. Token's position within exercise
4. Exercise's position within session
5. Client
6. Type of translation question
7. Seconds taken to answer question
8. Prompt
9. Other morphological features of the word
10. Tense
11. Person

The additional engineered features were:

1. Total number of tokens in exercise
2. Next token within exercise
3. Previous token within exercise
4. Days since last time token was encountered

5. Days since second last time token was encountered
6. Days since third last time token was encountered
7. Days since fourth last time token was encountered
8. Days since fifth last time token was encountered
9. Accuracy on last time token was encountered
10. Accuracy on second last time token was encountered
11. Accuracy on third last time token was encountered
12. Accuracy on fourth last time token was encountered
13. Accuracy on fifth last time token was encountered
14. Lowest average accuracy of surrounding tokens by same user (“hardest neighbor”)
15. Second lowest average accuracy of surrounding tokens by same user
16. Third lowest average accuracy of surrounding tokens by same user
17. Token length in characters

Appendix B: Decision trees fit on predicted values

Since the GBDT is an ensemble of iteratively optimized decision trees, no single tree can be visualized to summarize the predictive model. Such a visualization would be useful to gain additional insight in the machinations of the predictive model. A solution to arrive at a single visual summary is to fit a new decision tree on the *predicted* values using the input features as predictors. The resulting decision tree thus indicates which input features maximally differentiate the predicted values. In other words, using this approach, we can highlight which input features best explain the differences in the predictions made by each model, which provides a different lens through which to compare the two models. The two decision trees shown in Figure B1 are based on this approach. Their depths were determined by optimizing the approximation to the GBDT predictions using 5-fold cross-validation.

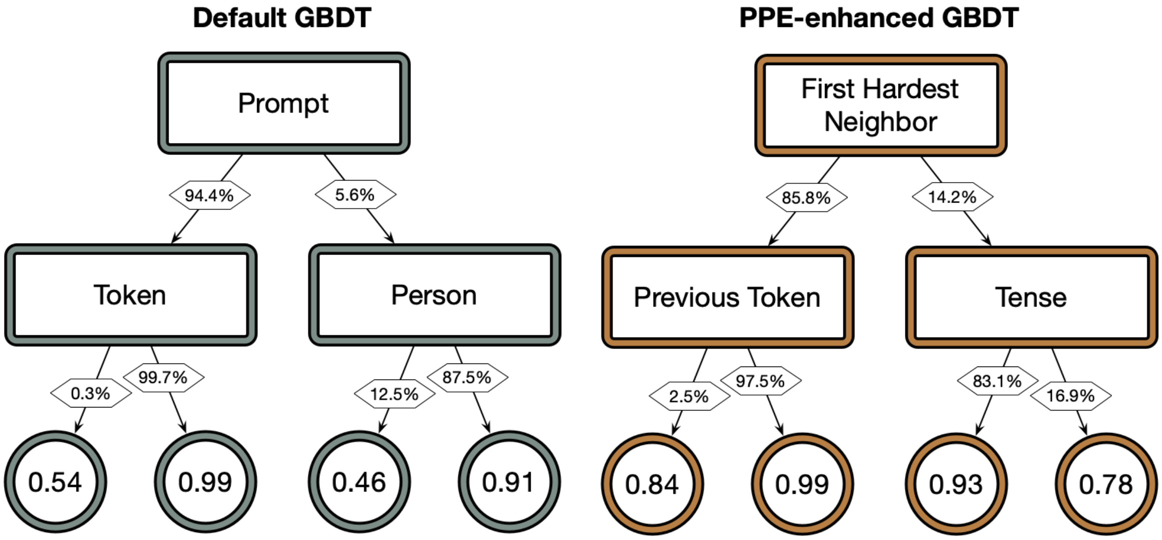

Figure B1. Cross-validated decision trees fit to the predictions made by the two models. The percentage of samples following a split at each node are shown in the diamond-shaped boxes; terminal nodes are round and indicate the proportion of correct responses predicted.

Figure B1 shows for both models which features are used to split the predicted values into the four most dominant values. Notably, both models have one dominant path that capture the majority—99.4% * 99.7% = 99.1% and 85.8% * 97.5% = 83.7% for the default and PPE-enhanced GBDT, respectively—of predicted values. Both of these majority paths have terminal nodes with predicted values of 0.99, indicating that predicted values are skewed towards very

high performance. Furthermore, the PPE-enhanced model generally predicts higher performance and neither model has a terminal node with predicted performance close to 0. This is not surprising since trees are only shown to a depth of two and the proportion of correct responses in the training set is 87.6%—a bias towards correct responses is to be expected. Despite the differences implied by predicted proportions in the terminal nodes, both models are very similar in terms of their true/false positive/negative rates (see the confusion matrices in Table B1) and AUCs (0.8530 versus 0.8538).

Table B1. Confusion matrices for the predictions made by the two models.

	Default GBDT predictions		PPE-enhanced GBDT predictions	
↓ True values	Correct	Incorrect	Correct	Incorrect
Correct	229,883<br>83.57%	6,041<br>2.2%	230,044<br>83.63%	5,880<br>2.14%
Incorrect	25,929<br>4.81%	13,230<br>4.81%	26,004<br>9.45%	13,155<br>4.78%

The most striking difference between the two models in Figure B1 is the lack of overlap between features the decision tree splits the predicted values on. The first split is made on a feature related to the context of the response that is predicted (the *prompt* or the *hardest neighbor*). The second split is made based on token identifiers (left branch; either the current or previous token) or a grammatical attribute (right branch; person or tense). One possible reason these features differ so drastically is that the full ensemble of trees used to make the predictions can utilize arbitrary and complex non-linear relationships between the various input features; the single decision trees fit to create the overview shown in Figure B1 cannot achieve the same. Hence, they likely pick up on different mechanics that the GBDTs that were used to make the predictions.
